# Postprandial Hypertriglyceridemia Predicts Development of Insulin Resistance Glucose Intolerance and Type 2 Diabetes

**DOI:** 10.1371/journal.pone.0145730

**Published:** 2016-01-25

**Authors:** Mohammad Aslam, Sarla Aggarwal, Krishna Kumar Sharma, Vikas Galav, Sri Venkata Madhu

**Affiliations:** 1 Center for Diabetes Endocrinology & Metabolism, Department of Medicine, University College of Medical Sciences (University of Delhi) & GTB Hospital, Delhi, India; 2 Department of Pathology, University College of Medical Sciences (University of Delhi) & GTB Hospital, Delhi, India; 3 Department of Pharmacology, University College of Medical Sciences (University of Delhi) & GTB Hospital, Delhi, India; The Ohio State University, UNITED STATES

## Abstract

Insulin resistance (IR) and type 2 diabetes mellitus (T2DM) have been found to be associated with postprandial hypertriglyceridemia (PPHTg). However, whether PPHTg can cause IR and diabetes is not clear. We therefore investigated the role of PPHTg in development of T2DM in rat model of T2DM. 96 male Wistar rats were randomized into four groups (24 rats each). Control Group A, high sucrose diet (HSD) Group B, HSD+Pioglitazone (10mg/kg/day) Group C and HSD+Atorvastatin (20mg/kg/day) Group D. Fat and glucose tolerance tests were done at regular intervals in all groups besides insulin and body weight measurement. At 26 weeks, low dose streptozotocin (15mg/kg,i.p.) was given to half of the rats. All rats were followed up till 48 weeks. PPHTg developed as early as week 2 in Group B and stabilized by week 14. Group B displayed highest PPHTg compared to other groups. Atorvastatin treatment (Group D) abolished PPHTg which became comparable to controls, pioglitazone treatment partially blunted PPHTg resulting in intermediate PPHTg. Group B with highest PPHTg showed highest subsequent IR, glucose intolerance (GI) and highest incidence of prediabetes at week 26 and diabetes at week 34 and 46 compared to other groups. Group D rats displayed lower IR, GI, low incidence of prediabetes and diabetes at these time points compared to Groups B and C. ROC analysis showed that triglyceride area under the curve of each time point significantly predicts the risk of diabetes. Present study provides the evidence that PPHTg predicts the development of IR, GI and T2DM in rat model of diet induced T2DM.

## Introduction

Postprandial hypertriglyceridemia (PPHTg) has emerged as an important independent risk factor for atherosclerosis particularly in patients with type 2 diabetes. Insulin resistance and type 2 diabetes mellitus have been reported to cause postprandial hypertriglyceridemia [1,2]. Whether PPHTg can lead to insulin resistance and type 2 diabetes is not known.

In recent years, some studies have shown that PPHTg is an early abnormality in the natural history of T2DM [1] and has been reported in newly detected type 2 diabetes patients, prediabetes subjects [3] as well as in genetically predisposed first degree relatives of type 2 diabetes patients [4]. These observations suggest that PPHTg may possibly be occurring much before the onset of T2DM. Short term experimental studies [5] and human case reports [6] also support our hypothesis that PPHTg could predict insulin resistance and glucose intolerance. The human evidence regarding the causal role of triglycerides in T2DM comes from the case report of triglyceride induced diabetes, where very high levels of triglycerides resulted in insulin resistance and diabetes and complete reversal of the overt diabetes and near complete reversal of insulin resistance occurred when lipid malabsorption was induced by bilio-pancreatic diversion which substantially lowered the triglyceride levels [6].

However, despite the growing evidence of a possible causal link between PPHTg and insulin resistance and T2DM, studies on its role in the pathogenesis of T2DM are few and preliminary. Further these studies were in patients with preexisting genetic defects which had resulted in extreme familial hypertriglyceridemia who had then developed glucose intolerance and diabetes [1,6]. Hence these results cannot be extrapolated to the general population.

In the present longitudinal experimental study we tested whether diet induced PPHTg in the absence of any genetic predisposition/specific genetic defects could lead to insulin resistance, glucose intolerance and type 2 diabetes mellitus. This we believe is the most common scenario of type 2 diabetes seen in clinical practice as specific genetic defects account for less than 5% of all type 2 diabetes cases [7]; therein lies the originality of this study.

The diet induced model of type 2 diabetes mellitus chosen for the study mimics and exhibits all the classical features that are known to occur in the natural history of human type 2 diabetes mellitus [8,9]. The rats chosen were wild wistar rats in whom no specific genetic predisposition is expected which is expected to provide evidence on any association between postprandial hypertriglyceridemia and diet induced glucose intolerance/diabetes. This could help to understand the precise role of postprandial hypertriglyceridemia in the pathogenesis of insulin resistance and type 2 diabetes mellitus.

## Materials and Methods

This study was approved by Institutional Ethics Committee—Animal Research (IEC-AR), University College of Medical Sciences, Delhi (IEC-AR approval letter no. UCMS/CAH/2010/82A-4, dated January 6, 2010). All the experiments were conducted in conformity with Public Health Service (PHS) policy and guidelines of Institutional Ethics Committee—Animal Research (IEC-AR), University College of Medical Sciences, Delhi. Ninety six selectively bred male Wistar rats (*Rattus norvegicus*) (6 weeks old) were procured from institutional central animal house facility. All the animals were housed under controlled environmental conditions (temp 20–25°C, humidity 50±20%) and diurnal cycle (12-h light/dark). After 2 weeks of acclimatization animals were randomized into stratified groups ensuring equal body weight means among them.

Ninety six male Wistar rats were randomized into four groups (24 rats each). Control group A was given standard chow diet, high sucrose diet group B, high sucrose diet + pioglitazone (10 mg/kg/day oral, USV pharmaceuticals) group C and high sucrose diet + atorvastatin (20 mg/kg/day oral, Zydus Cadila) group D. Group A and B were given vehicle (0.5% carboxymethyl cellulose) only. After overnight fasting (12 hour), oral fat challenge tests [10] at week 2,6,10,14,18,22,26,32,48 and oral glucose tolerance tests [11] at week 4,8,12,16,20,24,26,28,30,34 and 46 were done in all the four groups. In oral fat challenge test, 2 ml/kg olive oil was given orally following fasting blood sampling and then blood samples were collected from tail vein after 2, 4, 6 and 8 hours of olive oil loading. Similarly, for oral glucose tolerance test, 2 g/kg glucose was given orally following fasting blood sampling and then blood samples were collected from tail vein after 30, 60, 90 and 120 minutes of glucose loading. Body weight was recorded at the time of every fat challenge test and oral glucose tolerance test. Fasting serum insulin was also measured at the time of every fat challenge and oral glucose tolerance test. Fasting blood sugar levels were also measured at the time of every fat challenge test. At week 26, low dose streptozotocin (15 mg/kg, intraperitonial, Sigma chemicals) was given to half of the rats in each group (i.e., 12 rats in each group) to induce partial beta cell destruction [9] and an equal volume of vehicle (citrated buffer, pH 4.5) was given to remaining half rats in each of the four groups. At week 48, half of the rats from each group/subgroup were killed humanely using carbon dioxide inhalation under veterinary supervision. Visceral fat, subcutaneous fat and hepatic fat content were measured in killed rats at week 48 and pancreas were stored in 10% formalin for histopathology. Incidence of diabetes was also observed in remaining/surviving rats at week 72.

After two weeks of acclimatization i.e., day one of study, control group A was given control diet. Groups B, C and D were given high sucrose diet used by Rene et al. [8] with slight modification in view of the long term follow up period of 72 weeks. Experimental diet used in our study had 230 gm added sucrose per 1000 gm of diet. Sixty percent of total calorie intake was from carbohydrate source (including sucrose), 15% of total calorie intake was provided by protein and 25% by fat. All the rats were given their respective diet and water ad libitum unless specified.

Blood glucose was measured by glucometer (One Touch, Sure Step Life Scan, Johnson & Johnson). Triglyceride and total cholesterol in serum samples were measured by commercially available kits (Merck-Labkit, Spain, coefficient of variation for triglyceride and cholesterol was <1% within the assay and <2% between the assays) and HDLc was estimated by direct method (Accurex biomedicals, India, coefficient of variation <1% within the assay and <2% between the assays). Each time Quality control sera (BioRad, USA) were run along with unknown samples. The LDLc was calculated as following (Friedwald equation); LDL = [Total CHL–(HDL +TG/5)]. Serum insulin levels were measured by commercially available RIA kits (Millipore, USA, coefficient of variation <5% within the assay and <10% between the assays) and HOMA-IR was calculated as applicable to rats [12], HOMA-IR = [Fasting insulin (μU/ml) x Fasting glucose (mg/dl)]/2430.The cut offs of blood sugars values for diagnosis of prediabetes and diabetes are similar as applicable to humans [8,13] i.e., for diabetes; fasting blood sugar ≥7 mmol/L or 2 hour postprandial ≥11.1 mmol/L or both and for prediabetes fasting blood sugar 5.55 mmol/L mg/dl to<7 mmol/L mg/dl or 2 hour postprandial 7.77 mmol/L to <11.1 mmol/L or both.

Visceral fat was defined as the fat located inside the abdominal cavity and packed between the organs i.e., stomach, liver, intestines, kidneys. Visceral fat was excised and weighed immediately in sacrificed rats and calculated as Visceral fat % = (Excised visceral fat (gm) / Total body weight (gm)) x 100 [14,15]. Subcutaneous fat mass (SF) was defined as fat in the subcutaneous area of the abdominal wall and calculated as Subcutaneous Fat % = (Subcutaneous fat (gm) / Total body weight (gm)) x 100[14,15].

Hepatic fat content was measured by Folch method [16], for this purpose, volume of tissue sample was computed on the assumption that the tissue has the specific gravity of water i.e., the volume of 1 gm of tissue is 1 ml.

Pancreas were stored in 10% formalin until staining. Routine histopathology processing was done and obtained sections were stained with haematoxylin and eosin staining. Islet cell mass was analyzed on light microscopy (binocular).

## Statistical Analysis

Area under the curve was calculated by trapezoidal rule. To compare the variables viz lipid parameters, glucose intolerance, insulin, insulin resistance and body weight between the groups and within the groups at different time points, generalized estimating equation followed by least significant difference pair wise comparisons were applied using SPSS 20.0. Comparison of incident diabetes and prediabetes between the groups was done by Chi-square test. Receiver operating characteristic (ROC) curve analysis was used to evaluate the validity of postprandial triglyceride for predicting the risk of diabetes. Values were considered significant if P = <0.05.

## Results

All the parameters at baseline were found to be similar between the control group A and each of the three groups given a high sucrose diet.

Postprandial triglyceride area under the curve (PPTg—AUC) was observed to be significantly higher in high sucrose diet (HSD) fed rats as early as week 2 which stabilized by week 14 and remained high till week 48 as compared to the control group (Table 1). Postprandial triglyceride responses as indicated by PPTg—AUC to fat challenge in HSD fed rats were significantly blunted both by atorvastatin and pioglitazone (Table 1). With atorvastatin the blunting occurred after week 2 and was nearly complete approaching control values for most of the study till week 48 (Table 1). However, with pioglitazone the blunting occurred only after 10 weeks and remained significant for remainder of the study but was less complete when compared to atorvastatin at most of the time points (Table 1).

**Table 1:** Triglyceride area under the curves in all the four groups at different time points.

*Time points (week)*	*Group A Mean±SD (mmol L^-1^ 8 hr) (n = 24*, *25%)*	*Group B Mean±SD (mmol L^-1^ 8 hr) (n = 24*, *25%)*	*Group C Mean±SD (mmol L^-1^ 8 hr) (n = 24*, *25%)*	*Group D Mean±SD (mmol L^-1^ 8 hr) (n = 24*, *25%)*	*Significance*
*2*	*11*.*55±2*.*50*	*15*.*71±2*.*84*	*15*.*95±2*.*57*	*14*.*17±2*.*13*	*a = <0*.*001*, *b = <0*.*001*, *c = <0*.*001*, *d = ns*, *e = 0*.*03*, *f = 0*.*01*
*6*	*12*.*85±1*.*64*	*16*.*31±4*.*20*	*16*.*66±5*.*67*	*13*.*59±3*.*37*	*a = <0*.*001*, *b = 0*.*002*, *c = ns*, *d = ns*, *e = 0*.*01*, *f = 0*.*02*
*10*	*15*.*83±3*.*84*	*22*.*19±5*.*22*	*17*.*87±4*.*95*	*15*.*49±3*.*77*	*a = <0*.*001*, *b = ns*, *c = ns*, *d = 0*.*005*, *e = <0*.*001*, *f = ns*
*14*	*16*.*91±6*.*14*	*24*.*10±3*.*98*	*20*.*04±5*.*72*	*16*.*49±3*.*95*	*a = < 0*.*001*, *b = ns*, *c = ns*, *d = 0*.*006*, *e = <0*.*001*, *f = 0*.*01*
*18*	*17*.*41±5*.*18*	*26*.*70±6*.*16*	*22*.*52±5*.*50*	*17*.*97±3*.*82*	*a = <0*.*001*, *b = 0*.*001*, *c = ns*, *d = 0*.*01*, *e = <0*.*001*, *f = 0*.*001*
*22*	*18*.*59±7*.*61*	*27*.*05±3*.*69*	*21*.*65±3*.*80*	*18*.*21±2*.*68*	*a = <0*.*001*, *b = ns*, *c = ns*, *d = <0*.*001*, *e = <0*.*001*, *f = <0*.*001*
*26*	*18*.*25±2*.*88*	*28*.*35±4*.*50*	*21*.*97±3*.*40*	*19*.*04±3*.*70*	*a = <0*.*001*, *b = <0*.*001*, *c = ns*, *d = <0*.*001*, *e = <0*.*001*, *f = 0*.*006*
*32*	*17*.*86±3*.*78*	*28*.*95±4*.*16*	*18*.*76±3*.*86*	*17*.*85±3*.*87*	*a = <0*.*001*, *b = ns*, *c = ns*, *d = <0*.*001*, *e = <0*.*001*, *f = ns*
*48*	*14*.*01±2*.*89*	*27*.*84±2*.*58*	*18*.*52±5*.*23*	*14*.*81±4*.*27*	*a = <0*.*001*, *b = 0*.*001*, *c = ns*, *d = <0*.*001*, *e = <0*.*001*, *f = 0*.*01*

a = Group A vs Group B, b = Group A vs Group C, c = Group A vs Group D, d = Group B vs Group C, e = Group B vs Group D, f = Group C vs Group D

From these observations it can be concluded that postprandial triglyceridaemic burden over the follow up period of the study was lowest in control group A and atorvastatin treated HSD fed rats (group D). Postprandial triglyceridaemic burden over the follow up period of the study was highest in HSD fed rats given no intervention (group B). Postprandial triglyceridaemic burden in pioglitazone treated HSD fed rats (group C) was lower than group B but slightly higher than groups A and D. These observations are also applicable for peak triglyceride levels which were found to be significantly higher in group B compared to group A and group D at all time points. Peak triglyceride levels were also significantly higher in group B compared to group A at all time points (Table 2). Fasting triglyceride (FTG) levels were found to be normal in group B till week 10 even as significant postprandial hypertriglyceridemia was well documented from as early as second week. However, after week 10 FTG levels were also found to be significantly higher in group B compared to group A till week 48 (P = <0.05).

**Table 2:** Peak triglyceride levels during fat challenge test in all the four groups at different time points.

*Time points (week)*	*Group A Mean±SD (mmol L^-1^) (n = 24*, *25%)*	*Group B Mean±SD (mmol L^-1^) (n = 24*, *25%)*	*Group C Mean±SD (mmol L^-1^) (n = 24*, *25%)*	*Group D Mean±SD (mmol L^-1^) (n = 24*, *25%)*	*Significance*
*2*	*2*.*26±0*.*84*	*3*.*34±1*.*13*	*3*.*18±0*.*65*	*2*.*62±0*.*56*	*a = 0*.*002*, *b = 0*.*02*, *c = 0*.*01*, *d = ns*, *e = 0*.*03*, *f = 0*.*008*
*6*	*2*.*26±0*.*47*	*2*.*85±0*.*65*	*3*.*31±0*.*59*	*2*.*23±0*.*71*	*a = 0*.*02*, *b = 0*.*01*, *c = ns*, *d = 0*.*02*, *e = 0*.*005*, *f = 0*.*02*
*10*	*3*.*09±1*.*23*	*3*.*69±1*.*03*	*3*.*18±1*.*03*	*2*.*60±0*.*77*	*a = 0*.*02*, *b = ns*, *c = 0*.*04*, *d = 0*.*04*, *e = <0*.*001*, *f = 0*.*02*
*14*	*3*.*29±1*.*53*	*4*.*16±0*.*86*	*3*.*87±1*.*62*	*2*.*62±0*.*73*	*a = 0*.*01*, *b = 0*.*03*, *c = 0*.*04*, *d = ns*, *e = <0*.*001*, *f = 0*.*003*
*18*	*3*.*39±1*.*09*	*4*.*59±1*.*19*	*4*.*09±1*.*14*	*2*.*90±0*.*68*	*a = 0*.*009*, *b = 0*.*03*, *c = 0*.*04*, *d = ns*, *e = <0*.*001*, *f = 0*.*002*
*22*	*3*.*83±1*.*39*	*4*.*28±0*.*60*	*3*.*85±0*.*66*	*3*.*23±0*.*60*	*a = 0*.*04*, *b = ns*, *c = 0*.*02*, *d = ns*, *e = 0*.*001*, *f = 0*.*01*
*26*	*2*.*32±0*.*63*	*4*.*62±0*.*75*	*3*.*43±0*.*52*	*3*.*43±0*.*74*	*a = <0*.*001*, *b = <0*.*001*, *c = 0*.*002*, *d = 0*.*004*, *e = 0*.*003*, *f = ns*
*32*	*2*.*66±0*.*67*	*4*.*63±0*.*72*	*3*.*10±0*.*60*	*2*.*93±0*.*47*	*a = <0*.*001*, *b = 0*.*04*, *c = ns*, *d = <0*.*001*, *e = 0*.*001*, *f = ns*
*48*	*2*.*34±0*.*69*	*4*.*40±0*.*56*	*2*.*36±0*.*83*	*2*.*48±0*.*78*	*a = 0*.*007*, *b = ns*, *c = ns*, *d = 0*.*005*, *e = 0*.*009*, *f = ns*

a = Group A vs Group B, b = Group A vs Group C, c = Group A vs Group D, d = Group B vs Group C, e = Group B vs Group D, f = Group C vs Group D

We did not observe any significant trend in any of the other lipids (total cholesterol, HDL and LDL) in fasting and postprandial state throughout the study.

**Fig 1:**
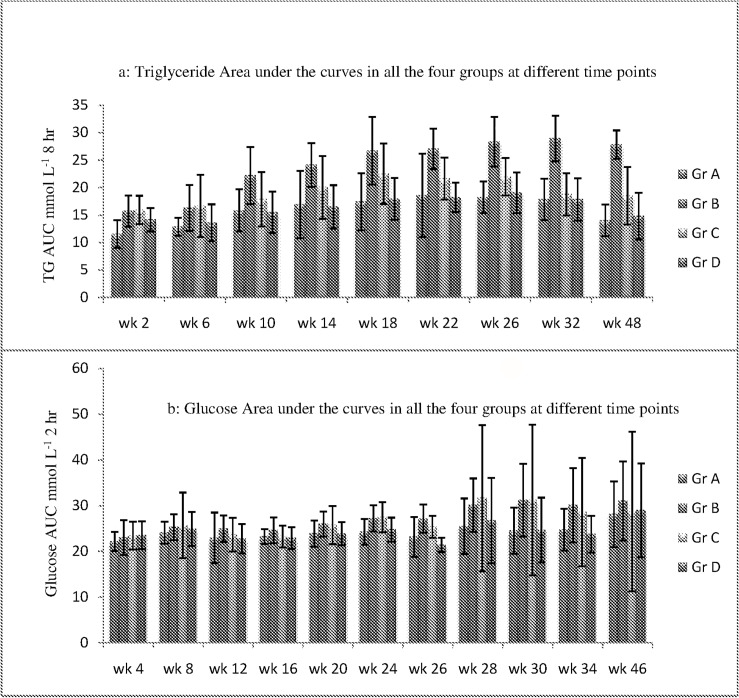
Triglyceride and glucose AUCs in all the four groups at different time points.

Glucose area under the curve was not significantly different from baseline until week 12 in any of the four groups studied. Glucose area under the curves were observed to be significantly higher overall in high sucrose diet fed rats compared to controls from week 12 to week 24 and 26. There were no significant differences in glucose area under the curves between atorvastatin treated rats and controls in the same period. In the pioglitazone treated rats glucose area under the curve was observed to be similar to controls at week 12. However, it was found to be higher at week 24 and 26 as compared to controls (Table 3). Overall, glucose—area under the curve was highest in high sucrose diet fed group when compared with (i) standard chow diet fed rats, (ii) high sucrose diet + Atorvastatin treated rats and (iii) high sucrose diet + Pioglitazone treated rats. Also, it has been observed that triglyceride area under the curves and glucose area under the curves were highest in high sucrose diet group B rats followed by pioglitazone treated group C compared to groups A and D throughout the study period (Fig 1A and 1B).

**Table 3:** Glucose area under the curves in all the four groups at different time points.

*Time points (week)*	*Group A Mean±SD (mmol L^-1^ 2 hr) (n = 24*, *25%)*	*Group B Mean±SD (mmol L^-1^ 2 hr) (n = 24*, *25%)*	*Group C Mean±SD (mmol L^-1^ 2 hr) (n = 24*, *25%)*	*Group D Mean±SD (mmol L^-1^ 2 hr) (n = 24*, *25%)*	*Significance*
*4*	*22*.*21±2*.*10*	*23*.*06±3*.*81*	*23*.*46±3*.*04*	*23*.*57±3*.*01*	*a = ns*, *b = ns*, *c = ns*, *d = ns*, *e = ns*, *f = ns*
*8*	*24*.*12±2*.*41*	*25*.*31±2*.*81*	*25*.*72±7*.*17*	*24*.*93±3*.*70*	*a = ns*, *b = <0*.*001*, *c = ns*, *d = ns*, *e = ns*, *f = ns*
*12*	*22*.*99±5*.*54*	*25*.*01±2*.*89*	*23*.*70±3*.*66*	*22*.*81±3*.*21*	*a = <0*.*001*, *b = ns*, *c = ns*, *d = 0*.*005*, *e = <0*.*001*, *f = ns*
*16*	*23*.*26±1*.*61*	*24*.*66±2*.*83*	*23*.*28±2*.*37*	*22*.*96±2*.*38*	*a = 0*.*04*, *b = ns*, *c = ns*, *d = ns*, *e = 0*.*04*, *f = ns*
*20*	*23*.*94±2*.*86*	*26*.*00±2*.*76*	*25*.*76±4*.*16*	*23*.*93±2*.*52*	*a = 0*.*01*, *b = ns*, *c = ns*, *d = ns*, *e = 0*.*02*, *f = ns*
*24*	*24*.*28±2*.*81*	*27*.*24±2*.*89*	*27*.*50±3*.*28*	*24*.*79±2*.*62*	*a = <0*.*001*, *b = <0*.*001*, *c = ns*, *d = ns*, *e = 0*.*003*, *f = 0*.*01*
*26*	*23*.*18±4*.*38*	*27*.*18±3*.*09*	*25*.*42±2*.*41*	*21*.*44±1*.*54*	*a = <0*.*001*, *b = 0*.*02*, *c = ns*, *d = 0*.*003*, *e = <0*. *001*, *f = <0*.*001*
*28*	*25*.*54±6*.*07*	*30*.*12±5*.*84*	*31*.*65±16*.*01*	*26*.*76±9*.*30*	*a = 0*.*002*, *b = ns*, *c = ns*, *d = ns*, *e = ns*, *f = ns*
*30*	*24*.*57±5*.*04*	*31*.*24±7*.*95*	*31*.*25±16*.*45*	*24*.*70±7*.*08*	*a = 0*.*01*, *b = ns*, *c = ns*, *d = ns*, *e = <0*.*001*, *f = ns*
*34*	*24*.*75±4*.*59*	*30*.*12±8*.*10*	*28*.*63±11*.*84*	*23*.*81±4*.*01*	*a = 0*.*01*, *b = ns*, *c = ns*, *d = ns*, *e = <0*.*001*, *f = ns*
*46*	*28*.*13±7*.*2*	*31*.*08±8*.*63*	*28*.*71±17*.*44*	*29*.*00±10*.*23*	*a = ns*, *b = ns*, *c = ns*, *d = ns*, *e = ns*, *f = ns*

a = Group A vs Group B, b = Group A vs Group C, c = Group A vs Group D, d = Group B vs Group C, e = Group B vs Group D, f = Group C vs Group D

Significantly higher (82.6%; 19/23) rats in high sucrose diet group B and 70.8% (17/24) pioglitazone treated rats in group C had impaired glucose tolerance (prediabetes) compared to control group A, 4.2% (1/24) at week 26 (P = <0.001 for groups B vs. A and groups C vs. A). Also, incidence of prediabetes in atorvastatin treated group D (12.5%; 3/24) was significantly lower compared to group B (P = <0.001) and group C (P = <0.001). The incidence of prediabetes was not significantly different between groups B and C (P = 0.54) and groups D and A (P = 0.60) at week 26.

Cumulative incidence of glucose intolerance (prediabetes+ diabetes) was significantly higher (100%; 23/23) in rats fed on high sucrose diet group B and pioglitazone treated rats in group C (83.33%; 20/24) compared to control group A (41.66%; 10/24) at week 34 (P = 0.001 for groups B vs. A and P = 0.007 for groups C vs. A). Also, incidence of glucose intolerance in atorvastatin treated group D (50%; 12/24) was significantly lower compared to group B (P = <0.001) and group C (P = 0.03). The incidence of glucose intolerance was not significantly different between groups B and C (P = 0.12) and group D and A (P = 0.77).

Similar trend was also observed at week 46 where incidence of glucose intolerance (prediabetes + diabetes) was significantly higher (100%; 23/23) in rats fed on high sucrose diet group B and pioglitazone treated rats in group C (87.5%; 21/24) compared to control group A, (54.16%; 13/24) (P = <0.001 for groups B vs. A and P = 0.02 for groups C vs. A). Also, incidence of glucose intolerance in atorvastatin treated group D (58.33%; 14/24) was significantly lower compared to group B (P = <0.001) and group C (P = <0.05). The incidence of glucose intolerance was not significantly different between groups B and C (P = 0.24) and groups D and A (P = 0.77).

No rat developed overt diabetes in any of the four groups till week 26. At week 34 i.e., 4 weeks after low dose of streptozotocin, 90.90% (10/11) of rats in high sucrose diet group B and 33.33% (4/12) rats in pioglitazone treated group C developed diabetes as compared to only 27.27% (3/11) in control group A (P = 0.009 for groups B vs. A and P = 0.75 for groups C vs. A). Only 25% (3/12) rats in atorvastatin treated group D developed diabetes at week 34 which was lower than control group A (P = 0.90) and pioglitazone treated group C (P = 0.65). The incidence of diabetes in atorvastatin treated group D was significantly lower compared to group B (P = 0.005). Also, incidence of diabetes in group B was significantly higher than group C (P = 0.01). These figures broadly reflected the distribution of prediabetes immediately preceding streptozotocin injection.

The cumulative incidence of diabetes increased further reaching 100% (11/11) in high sucrose diet group B at week 46 which was significantly higher compared to control group A 45.45% (5/11) (P = 0.01). The cumulative incidence of diabetes at week 46 was found to be 50% (6/12) in pioglitazone treated group C and 41.66% (5/12) in atorvastatin treated group D which were both significantly lower than in group B (P = 0.02 for groups C vs. B and P = 0.009 for groups D vs. B). A similar trend of cumulative diabetes incidence was also observed at week 72 in the remaining rats. Even in rats not given streptozotocin ‘STZ non-treated’ about 25% developed diabetes in group B whereas none of the rats in the other groups developed diabetes by week 72.

**Fig 2:**
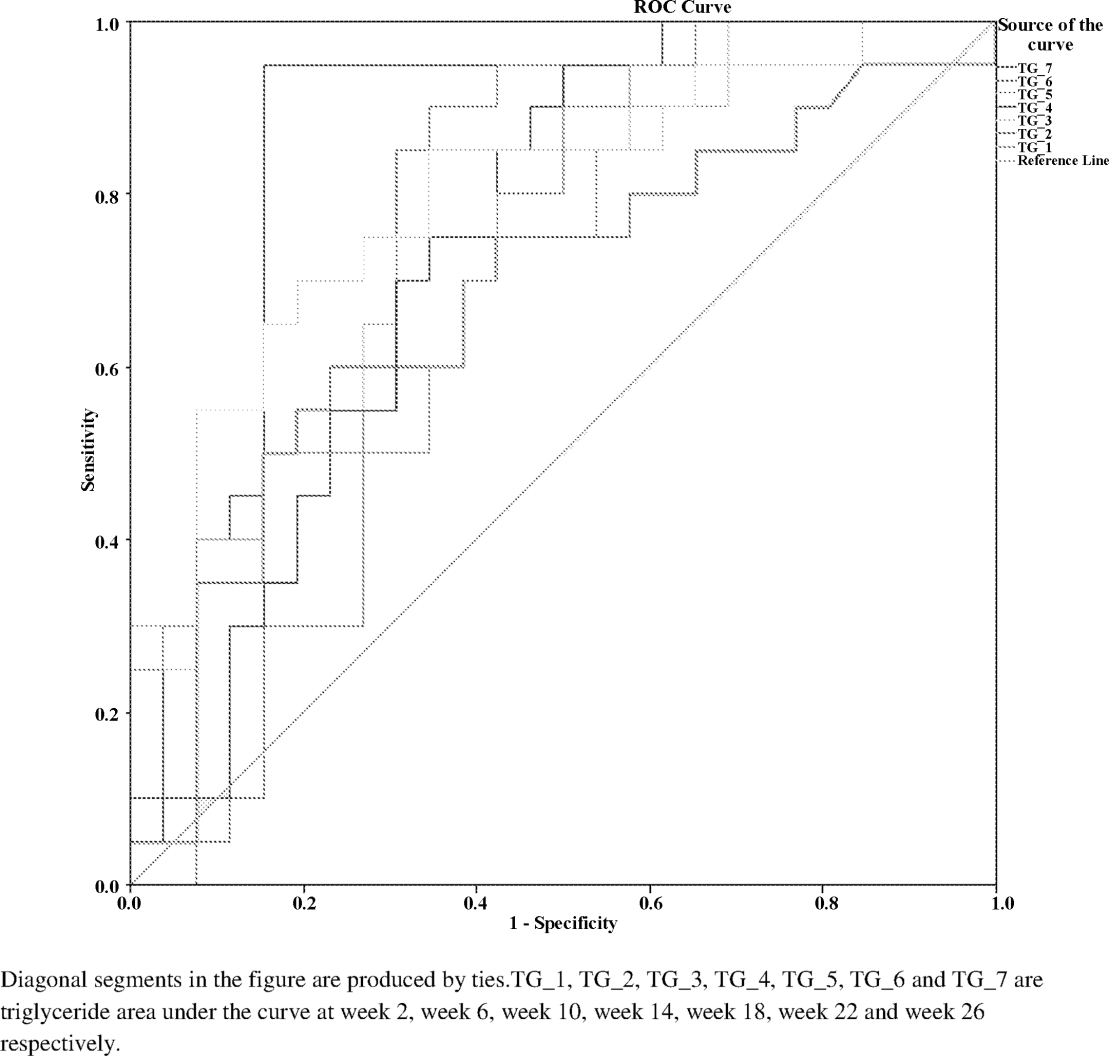
Receiver operating characteristic (ROC) curve plots for TG-AUC predicting diabetes risk.

The above findings suggest that high sucrose diet fed group B rats which displayed the highest postprandial triglyceride burden also had highest incidence of diabetes in them. The control group as well as atorvastatin treated group which had the least postprandial triglyceride burden also had the lowest incidence of diabetes in them. The pioglitazone treated group with intermediate postprandial triglyceride burden had diabetes developing in an intermediate number higher than control and atorvastatin treated groups. Further, receiver operating characteristic (ROC) curves plotted for triglyceride area under the curve for group B after adjustment for effect of high sucrose diet passed through the upper left corner (Fig 2). This analysis showed that triglyceride area under the curve of each time point significantly predicted the risk of diabetes. Of all these time points, triglyceride area under the curve of week 26 was the strongest predictor of diabetes.

**Fig 3:**
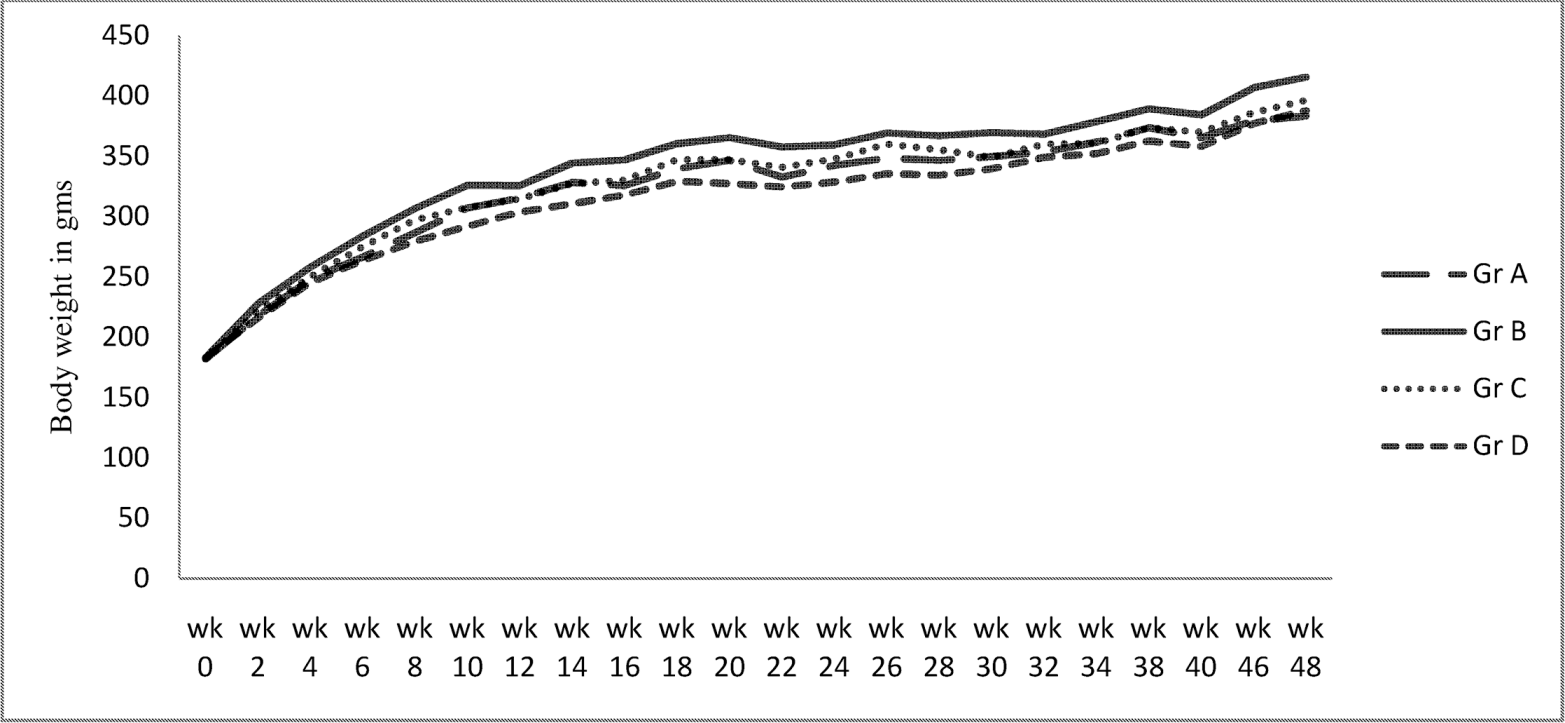
Body weight in all the four groups at different time points.

There was a progressive increase in body weight throughout the study in all the four groups with a greater part of weight gain occurring by week 18. Rats in high sucrose diet group displayed significantly higher body weight at all time points after week 6 compared to controls or the atorvastatin treated group. Pioglitazone treated group had intermediate weight gain which was higher though statistically insignificant than controls and atorvastatin treated rats (Fig 3) (S1 Table).

Visceral fat content in rats sacrificed at week 48 was found to be significantly higher in all the three experimental groups fed with high sucrose diet as compared to controls. However, subcutaneous fat content was similar in all the four groups. Hepatic fat content was found to be significantly lower in atorvastatin treated group D compared group B and C whereas control group A had intermediate hepatic fat content (Table 4).

**Table 4:** Visceral fat, hepatic fat and subcutaneous fat contents in rats killed at week 48.

*Parameters*	*Group A Mean±SD (n = 8)*	*Group B Mean±SD (n = 9)*	*Group C Mean±SD (n = 10)*	*Group D Mean±SD (n = 9)*	*significance*
*Hepatic Fat (mg/gm of tissue)*	*45*.*70±10*.*06*	*51*.*62±11*.*04*	*47*.*49±11*.*13*	*34*.*72±14*.*32*	*a = ns*, *b = ns*, *c = ns*, *d = ns*, *e = 0*.*01*, *f = 0*.*04*
*Visceral Fat (%)*	*4*.*60±0*.*97*	*6*.*82±2*.*01*	*6*.*86±1*.*22*	*6*.*06±1*.*12*	*a = 0*.*01*, *b = <0*.*001*, *c = 0*.*01*, *d = ns*, *e = ns*, *f = ns*
*Subcutaneous Fat (%)*	*3*.*27±0*.*89*	*3*.*30±0*.*76*	*3*.*22±0*.*72*	*3*.*44±0*.*58*	*a = ns*, *b = ns*, *c = ns*, *d = ns*, *e = ns*, *f = ns*

a = Group A vs Group B, b = Group A vs Group C, c = Group A vs Group D, d = Group B vs Group C, e = Group B vs Group D, f = Group C vs Group D

**Fig 4:**
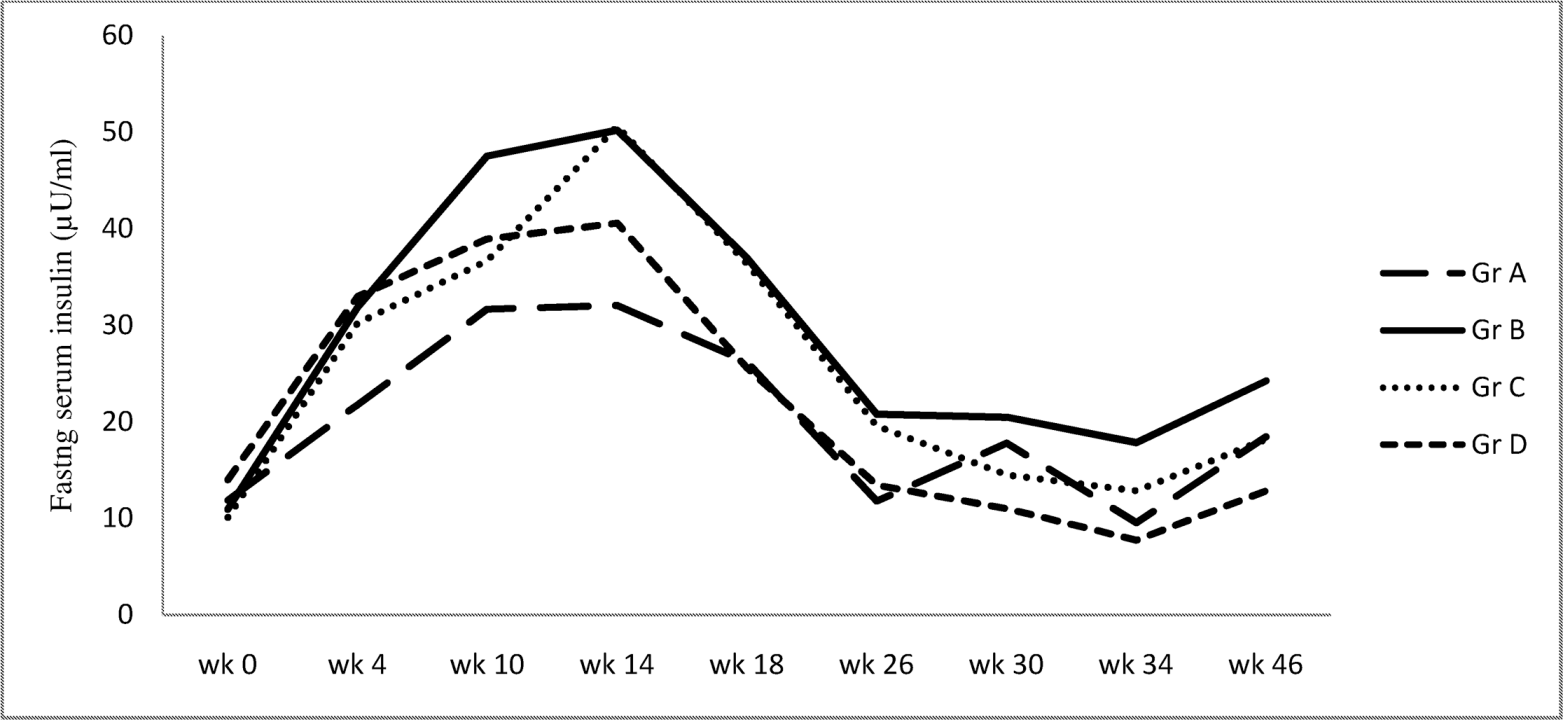
Fasting serum insulin levels at various time points.

In control group fasting insulin levels increased from baseline till week 10, remained there till week 14 and then showed a slow decline through week 18 and 22 till week 26 when low dose streptozotocin was given. This pattern which was observed in all the four groups, may represent age related effects on insulin levels. Fasting insulin levels were significantly higher in high sucrose diet group compared to controls from week 4 till week 26. Similar trend was observed in pioglitazone and atorvastatin treated groups. Although this was significant only with pioglitazone treated group at week 14 and week 26 (Fig 4) (S2 Table).

**Fig 5:**
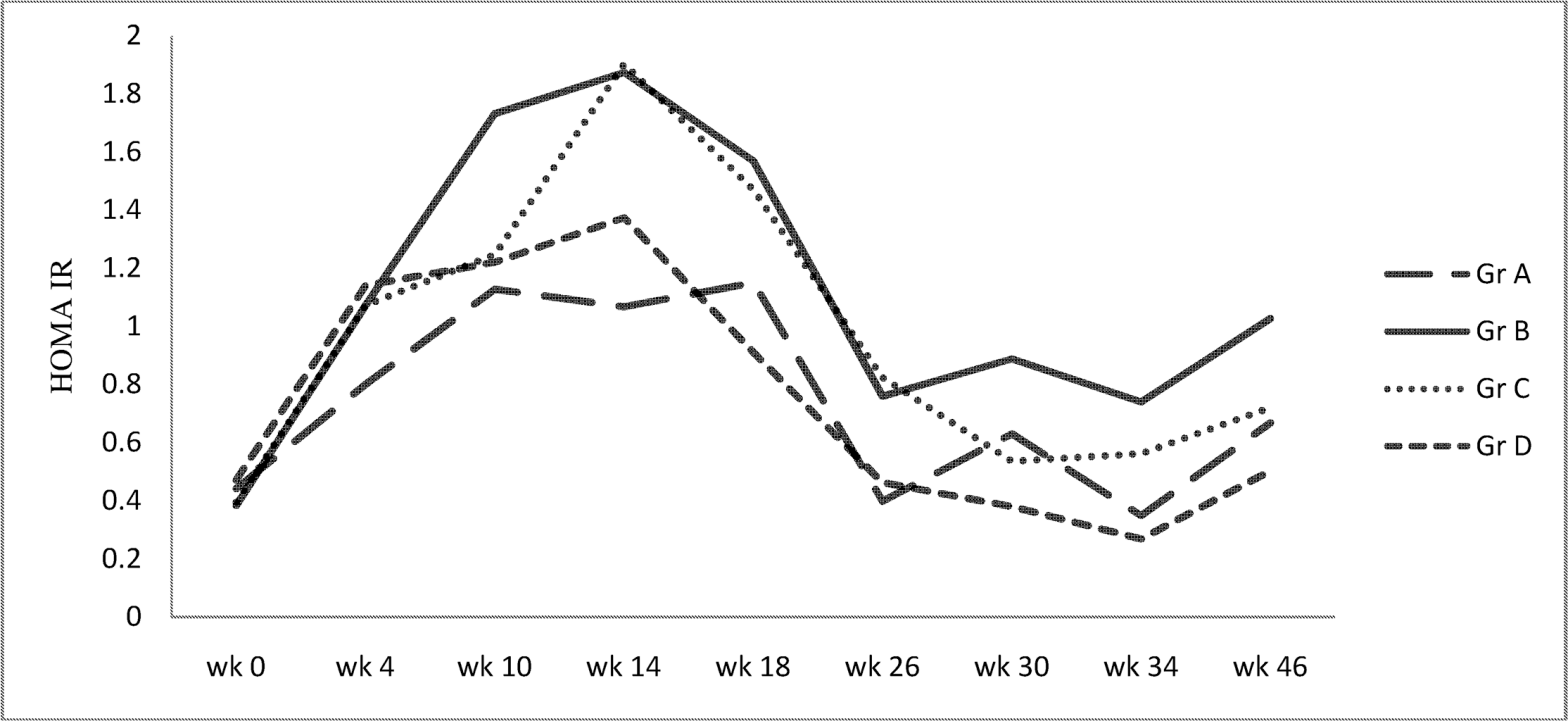
HOMA-IR in all the four groups at various time points.

Insulin resistance indicated by HOMA-IR was significantly higher in high sucrose diet group versus control by week 10, and also in pioglitazone and atorvastatin treated groups by week 14. HOMA-IR remained significantly higher in high sucrose diet fed group and pioglitazone treated group compared to controls till week 26. However, HOMA-IR returned to control levels by week 26 in atorvastatin treated group (Fig 5) (S3 Table). Also, postprandial triglyceride area under the curve of week 10 correlated positively with HOMA-IR of week 18 (r = 0.479, P = 0.01) in high sucrose diet fed group B.

Histopathology of pancreas in all the four groups revealed no significant difference in beta cell mass or structure between the groups.

## Discussion

The present study demonstrates that wild male Wistar rats fed high sucrose diet develop significant postprandial hypertriglyceridemia after fat challenge by 2nd week of sucrose feeding when compared with those fed standard chow diet and continue to display a significantly higher postprandial hypertriglyceridemic burden thereafter. These rats also start displaying significant insulin resistance from week 10, significantly higher glucose intolerance indicated by glucose area under the curve from week 12 and a significantly higher incidence of prediabetes by week 26 and overt type 2 diabetes by week 34. Furthermore, insulin resistance, glucose area under the curve, subsequent incident prediabetes and incident diabetes was highest in high sucrose diet fed rats who also had the greatest preceding postprandial triglyceride burden.

The demonstration of postprandial hypertriglyceridemia early in this study even when rats still displayed normal glucose tolerance is in agreement with an earlier study which reported that postprandial hypertriglyceridemia was prevalent in first-degree relatives of type 2 diabetes patients despite normal fasting triglyceride levels and normal glucose tolerance [17]. It would thus appear that whether it results from genetic predisposition in first degree relatives of diabetes patients or from dietary influences in the form of high sucrose diet noted in our study in rats, postprandial hypertriglyceridemia may play a key role in the development of insulin resistance and consequent glucose intolerance. It is possible that gene-environment interaction between postprandial hypertriglyceridemia associated genes and environmental factors such as dietary carbohydrate and fat consumption may result in a cascading effect on incident glucose intolerance.

The sequence of events viz development of postprandial hypertriglyceridemia very early, followed by insulin resistance and glucose intolerance, provide clear evidence for the first time in a diet induced rat model of type 2 diabetes mellitus that postprandial hypertriglyceridemia predicts the development of insulin resistance and subsequent glucose intolerance. Further, ROC analysis also showed that triglyceride area under the curve of each time point significantly predicts the risk of diabetes in high sucrose diet group. These findings point to a critical role for postprandial hypertriglyceridemia in the pathogenesis of insulin resistance and glucose intolerance. However, studies on knockout rat models would be required to establish the pathogenic role of postprandial hypertriglyceridemia in the development of type 2 diabetes.

The main strength of the present study is that it is a long term follow up study of 48 weeks which allows inferences on cause effect relationship. Further, this is the first comprehensive study where postprandial hypertriglyceridemia has been studied so extensively in relation to insulin resistance and glucose intolerance. Careful designing of the study and the repeated measurements of key study parameters at regular intervals allowed us to address the key question of whether postprandial hypertriglyceridemia plays a role in the development of insulin resistance and glucose intolerance. Inclusion of two additional groups of high sucrose fed rats who were given either pioglitazone or atorvastatin allowed us to evaluate the role of postprandial hypertriglyceridemia with greater strength and clarity.

Pioglitazone and atorvastatin were chosen as both are known to reduce postprandial hypertriglyceridemia [10,18]. Further, the beneficial effects of pioglitazone have been primarily observed on triglycerides in both animal [19] and humans [20,21] with minimal effects on other lipid components [20,21]. Similarly, the lipid lowering effects of atorvastatin in rats and mice have consistently been documented only on triglycerides [10,22,23]. Also, in most studies statins do not decrease LDL levels in these animals. This is believed to be due to the fact that rodents transport most of their serum cholesterol in HDL fraction and not LDL fraction [24] suggesting profound effects of statins only on triglycerides in rats and mice.

Pioglitazone exerted its beneficial effects possibly by increasing triglyceride accumulation in adipose tissues and improving their metabolism in liver. Number of clinical as well as experimental studies have shown that treatment with pioglitazone reduces postprandial [18] as well as fasting triglyceride [25,26] levels. Further, it has been demonstrated that PPAR-γ agonists decrease the incidence of diabetes significantly when given to subjects with impaired glucose tolerance [27]. Atorvastatin is believed to accelerate remnants clearance through hepatic low density lipoprotein receptors resulting in a reduction in fasting and postprandial triglyceride levels [10,28,29].

In our study we observed significant blunting of postprandial hypertriglyceridemia with both pioglitazone and atorvastatin when they were co-administered with high sucrose diet. Progressive blunting of postprandial hypertriglyceridemia from partial to complete by pioglitazone and atorvastatin respectively led to a progressive reduction in insulin resistance and glucose intolerance observed in high sucrose diet fed rats. Atorvastatin group which had the least postprandial triglyceride burden also had the lowest glucose intolerance in subsequent weeks including week 26 and 34. Pioglitazone pretreated rats who had intermediate postprandial triglyceride burden showed intermediate glucose intolerance in subsequent weeks which was lower than that of high sucrose diet fed rats but higher than atorvastatin pretreated rat. These findings further reinforced the hypothesis that insulin resistance and glucose intolerance were indeed a consequence of postprandial hypertriglyceridemia. However, we cannot completely rule out other possible effects of these drugs like anti-inflammatory, antioxidant or other pleiotropic effects which also could have played a role in reduction of glucose intolerance.

It would thus appear that it is postprandial hypertriglyceridemia that predicts glucose intolerance and not vice versa. This hypothesis is further supported by an earlier experimental study which showed that blunting of postprandial lipaemia by dietary diacylglycerol in Otsuka Long-Evans Tokushima Fatty (OLETF) rats with overt type 2 diabetes resulted in improvement of lipid metabolism, glucose tolerance and retardation of diabetes progression [11]. Similarly, a case report demonstrated that surgical correction of extreme hypertriglyceridemia in two sisters were associated with improvements in insulin-stimulated glucose uptake, oxidation and storage with resultant reversal of diabetes [6], suggesting that insulin resistant diabetes can be caused by extremely high levels of triglyceride. An acute metabolic study in healthy subjects which showed a decrease in insulin sensitivity during postprandial lipaemia [30] further validates our hypothesis. Authors of this study suggested that decreased insulin sensitivity is a consequence of elevated plasma levels of triglyceride-rich lipoproteins independently of plasma NEFA levels and suggested that postprandial lipaemia could be the cause of insulin resistance [30].

Histopathological findings of pancreas in our study revealed no significant difference between standard chow diet fed rats and high sucrose diet fed rats which is in contrast to earlier study of Del Zotto and co-workers who reported increase in beta cell mass in high sucrose diet fed rats [31]. This discrepancy can be due to the following; in the long term study by Del Zotto and co-workers histology of the pancreas was performed in 38 week old rats after a 30 week follow up period at a stage when they were obese and had the metabolic syndrome. Frank glucose intolerance, prediabetes or diabetes was not reported in them although the random blood sugar was found to be higher than controls. At this stage beta cell mass is expected to be increased due to insulin resistance. On the other hand, in the present study we undertook histology of pancreas only in 56 week old rats involving 48 week follow up period. Further this was 14 weeks after the rats developed overt diabetes and 22 weeks after they were clearly prediabetic indicating that the rats in whom beta cell mass was assessed in our study had diabetes/prediabetes for a significant length of time prior to this assessment. Further these rats were also older. It is possible that beta cell mass might have increased earlier at a younger age in these rats at the stage of metabolic syndrome/insulin resistance and prediabetes at week 26. However, the beta cell mass would be expected to decrease in them with the onset of diabetes and thereafter [32]. This decline in beta cell mass after diabetes onset could explain the absence of a significant increase in beta cell mass in our study. However, it would be difficult to comment about the beta cell mass with certainty from the results as we did not use immunohistochemistry and this remains a limitation of our study.

In summary, the findings of the present study clearly show that the postprandial hypertriglyceridemia predicts the development of insulin resistance, glucose intolerance and subsequently diabetes in a rat model of type 2 diabetes mellitus. Further, findings of present study suggest that postprandial hypertriglyceridemia may be used as a biomarker for the prediction of risk of type 2 diabetes.

## Supporting Information

S1 Table Body weight in all the four groups at different time points. (DOCX)

S2 Table Fasting serum insulin levels in all the four groups at different time points. (DOCX)

S3 Table HOMA IR in all the four groups at different time points. (DOCX)

## Abbreviations

Gl-AUC: glucose area under the curve
IGT: impaired glucose tolerance
ns: not significant
OGTT: oral glucose tolerance test
PPHTg: postprandial hypertriglyceridemia
TG-AUC: triglyceride area under the curve
wk: week
