# Supplementary material for: Postprandial Hypertriglyceridemia Predicts Development of Insulin Resistance Glucose Intolerance and Type 2 Diabetes
Source: PLoS One. 2016 Jan 25;11(1):e0145730. doi: 10.1371/journal.pone.0145730 (PMC4725668; doi:10.1371/journal.pone.0145730)
Supplement: S1 Table — (DOCX) [file pone.0145730.s001.docx]

S1 Table. Body weight in all the four groups at different time points

| *Time points*  *(week)* | *Group A*  *Mean ± SD*  *(gm)* | *Group B*  *Mean ± SD*  *(gm)* | *Group C*  *Mean ± SD*  *(gm)* | *Group D*  *Mean ± SD*  *(gm)* | *Significance* |
| --- | --- | --- | --- | --- | --- |
| *0* | *182.17±13.14* | *182.83±15.41* | *182.17±13.03* | *183.04±13.75* | *a=ns, b=ns, c=ns, d=ns, e=ns, f=ns* |
| *2* | *217.63±19.84* | *227.63±18.67* | *222.08±20.19* | *216.08±18.79* | *a=ns, b=ns, c=ns, d=ns, e=0.02, f=ns* |
| *4* | *247.88±22.20* | *257.96±18.53* | *250.08±24.04* | *245.38±20.99* | *a=ns, b=ns, c=ns, d=ns, e=0.02, f=ns* |
| *6* | *266.50±25.42* | *283.54±21.27* | *275.04±26.21* | *263.67±21.84* | *a=0.01, b=ns, c=ns, d=ns, e=0.001, f=ns* |
| *8* | *286.71±23.26* | *306.67±21.59* | *297.33±30.28* | *279.50±24.79* | *a=0.002, b=ns, c=ns, d=ns, e=<0.001, f=0.02* |
| *10* | *307.12±27.09* | *325.88±28.73* | *307.50±32.25* | *292.25±26.25* | *a=0.01, b=ns, c=ns, d=0.03, e=<0.001, f=ns* |
| *12* | *315.33±26.51* | *325.71±25.94* | *314.67±33.15* | *303.75±28.47* | *a=ns, b=ns, c=ns, d=ns, e=0.004, f=ns* |
| *14* | *328.33±26.32* | *344.33±29.92* | *327.46±35.58* | *310.67±28.11* | *a=0.04, b=ns, c=0.02, d=ns, e=<0.001, f=ns* |
| *16* | *325.79±26.32* | *346.63±29.17* | *330.29±34.77* | *318.08±29.70* | *a=0.008, b=ns, c=ns, d=ns, e=0.001, f=ns* |
| *18* | *339.54±27.99* | *360.33±34.57* | *347.33±38.17* | *329.21±30.64* | *a=0.01, b=ns, c=ns, d=ns, e=0.001, f=ns* |
| *20* | *346.33±29.19* | *365.33±33.72* | *347.38±38.89* | *327.25±30.61* | *a=0.03, b=ns, c=0.02, d=ns, e=<0.001, f=0.04* |
| *22* | *332.75±28.32* | *357.61±34.66* | *340.87±38.14* | *324.54±28.30* | *a=<0.001, b=ns, c=ns, d=ns, e=<0.001, f=ns* |
| *24* | *342.25±28.98* | *359.35±33.84* | *347.75±35.98* | *328.71±28.02* | *a=ns, b=ns, c=ns, d=ns, e=0.001, f=0.03* |
| *26* | *348.35±31.96* | *369.04±35.65* | *360.25±34.36* | *335.46±27.28* | *a=0.03, b=ns, c=ns, d=ns, e=<0.001, f=0.005* |
| *28* | *346.32±31.25* | *366.91±36.44* | *355.33±31.80* | *334.22±28.02* | *a=0.03, b=ns, c=ns, d=ns, e=0.001, f=0.01* |
| *30* | *349.45±31.74* | *369.32±37.98* | *349.21±32.94* | *339.57±26.61* | *a=ns, b=ns, c=ns, d=ns, e=0.002, f=ns* |
| *32* | *353.38±31.71* | *368.05±39.40* | *360.14±37.16* | *349.27±27.70* | *a= ns, b= ns, c= ns, d= ns, e= ns, f= ns* |
| *34* | *361.53±33.67* | *378.65±41.67* | *360.85±39.68* | *352.36±27.12* | *a= ns, b= ns, c= ns, d= ns, e= 0.01, f= ns* |
| *46* | *379.22±34.55* | *406.60±48.00* | *386.25±49.63* | *377.14±37.86* | *a=0.03, b=ns, c=ns, d=ns, e=0.02, f=ns* |
| *48* | *383.28±38.47* | *415.45±52.78* | *396.40±51.12* | *387.91±37.71* | *a=0.02, b=ns, c=ns, d=ns, e=0.04, f=ns* |

a=Group A vs Group B, b=Group A vs Group C, c=Group A vs Group D, d=Group B vs Group C, e=Group B vs Group D, f=Group C vs Group D
