# Supplementary material for: Postprandial Hypertriglyceridemia Predicts Development of Insulin Resistance Glucose Intolerance and Type 2 Diabetes
Source: PLoS One. 2016 Jan 25;11(1):e0145730. doi: 10.1371/journal.pone.0145730 (PMC4725668; doi:10.1371/journal.pone.0145730)
Supplement: S2 Table — (DOCX) [file pone.0145730.s002.docx]

S2 Table. Fasting Serum Insulin levels in all the four groups at different time points

| *Time points*  *(week)* | *Group A*  *Mean ± SD*  *(µU/ml)* | *Group B*  *Mean ± SD*  *(µU/ml)* | *Group C*  *Mean ± SD*  *(µU/ml)* | *Group D*  *Mean ± SD*  *(µU/ml)* | *Significance* |
| --- | --- | --- | --- | --- | --- |
| *0* | *11.85±6.39* | *10.93±7.88* | *10.11±8.58* | *13.96±9.37* | *a=ns, b=ns, c=ns, d=ns, e=ns, f=ns* |
| *4* | *21.74±18.62* | *31.83±16.15* | *30.22±15.10* | *32.95±16.26* | *a=0.04, b=ns, c=0.02, d=ns, e=ns, f=ns* |
| *10* | *31.67±15.96* | *47.54±26.13* | *36.71±20.13* | *38.91±15.40* | *a=0.01, b=ns, c=ns, d=ns, e=ns, f=ns* |
| *14* | *32.05±15.07* | *50.21±23.07* | *50.61±31.63* | *40.58±18.53* | *a=0.001, b=0.008, c=ns, d=ns, e=ns, f=ns* |
| *18* | *26.18±15.05* | *36.98±28.80* | *36.40±23.37* | *25.58±11.14* | *a=0.04, b=ns, c=ns, d=ns, e=0.02, f=0.03* |
| *26* | *11.80±8.18* | *20.79±17.54* | *19.58±9.62* | *13.44±13.23* | *a=0.02, b=0.002, c=ns, d=ns, e=ns, f=ns* |
| *30* | *17.81±7.83* | *20.46±20.26* | *14.51±8.95* | *11.01±5.39* | *a=ns, b=ns, c=0.001, d=ns, e=0.03, f=ns* |
| *34* | *9.54±8.69* | *17.85±8.61* | *12.85±8.68* | *7.73±6.33* | *a=0.002, b=ns, c=ns, d=ns, e=<0.001, f=0.03* |
| *46* | *22.02±12.41* | *27.90±21.39* | *19.08±8.33* | *25.05±11.37* | *a=ns, b=ns, c=ns, d=ns, e=ns, f=0.04* |

a=Group A vs Group B, b=Group A vs Group C, c=Group A vs Group D, d=Group B vs Group C, e=Group B vs Group D, f=Group C vs Group D
