# Supplementary material for: Postprandial Hypertriglyceridemia Predicts Development of Insulin Resistance Glucose Intolerance and Type 2 Diabetes
Source: PLoS One. 2016 Jan 25;11(1):e0145730. doi: 10.1371/journal.pone.0145730 (PMC4725668; doi:10.1371/journal.pone.0145730)
Supplement: S3 Table — (DOCX) [file pone.0145730.s003.docx]

S3 Table. HOMA IR in all the four groups at different time points

| Time points  (week) | Group A  Mean±SD | Group B  Mean±SD | Group C  Mean±SD | Group D  Mean±SD | Significance |
| --- | --- | --- | --- | --- | --- |
| 0 | 0.44±0.23 | 0.38±0.25 | 0.39±0.36 | 0.47±0.31 | *a=ns, b=ns, c=ns, d=ns, e=ns, f=ns* |
| 4 | 0.80±0.72 | 1.07±0.55 | 1.07±0.62 | 1.14±0.55 | *a=ns, b=ns, c=ns, d=ns, e=ns, f=ns* |
| 10 | 1.12±0.59 | 1.73±1.13 | 1.24±0.67 | 1.22±0.52 | a=0.01, b=ns, c=ns, d=ns, e=0.04, f=ns |
| 14 | 1.06±0.54 | 1.87±0.90 | 1.90±1.31 | 1.37±0.62 | a=0.003, b=0.002, c=0.03, d=ns, e=0.02, f=ns |
| 18 | 1.14±0.69 | 1.56±1.04 | 1.46±1.18 | 0.91±0.39 | a=0.03, b=ns, c=0.02, d=ns, e=0.003, f=0.02 |
| 26 | 0.39±0.31 | 0.75±0.63 | 0.82±0.43 | 0.46±0.45 | a=0.01, b=<0.001, c=ns, d=ns, e=ns, f=0.004 |
| 30 | 0.63±0.31 | 0.88±0.74 | 0.53±0.33 | 0.37±0.20 | a=ns, b=ns, c=0.001, d=ns, e=0.02, f=ns |
| 34 | 0.34±0.30 | 0.73±0.37 | 0.56±0.37 | 0.26±0.22 | a=<0.001, b=0.04, c=ns, d=ns, e=<0.001, f=0.002 |
| 46 | 0.86±0.53 | 1.16±1.00 | 0.76±0.33 | 0.98±0.45 | *a=ns, b=ns, c=ns, d=ns, e=ns, f=ns* |

a=Group A vs Group B, b=Group A vs Group C, c=Group A vs Group D, d=Group B vs Group C, e=Group B vs Group D, f=Group C vs Group D
